# The nasal microbiome of predicting bronchopulmonary dysplasia in preterm infants

**DOI:** 10.1038/s41598-022-10770-3

**Published:** 2022-05-11

**Authors:** Yanping Xu, Yeqing Huang, Zhen Shen, Liping Shi

**Affiliations:** 1 NICU, The Children’s Hospital, Zhejiang University School of Medicine, Hangzhou, China; 2 National Clinical Research Center for Child Health, 3333 Binsheng Road, Hangzhou, China; 3 Central Laboratory, The Children’s Hospital, Zhejiang University School of Medicine, Hangzhou, China

**Keywords:** Predictive markers, Biomarkers, Paediatric research, Biomarkers, Medical research

## Abstract

Bronchopulmonary dysplasia (BPD) is a chronic lung disease of prematurity and may cause substantial long-term disabilities. To characterize and compare the nasal swabs microbiome of early stage in premature infants and determine whether microbial diversity or composition in the nostrils associated with BPD disease. We performed a prospective observational cohort design. Preterm neonates less than or equal to 30 weeks of gestation were recruited from NICU, Children's Hospital, Zhejiang University School of Medicine from 2019 to 2020. Sterile foam swabs were collected from anterior nares at 1 and 3 weeks of postnatal age. We used PCR amplification and 16S rDNA sequencing. Neonatal demographic data including gestational age, birth weight, medication administration history and discharge outcomes were recorded. A total of 49 nasal swab samples were collected from 28 premature infants. Thirteen infants with BPD and 15 controls were finally involved in the study. Birth weights ranged from 700 to 1550 g. Gestational age ranged from 25^2/7^ to 30. We found increased in the expression of *Prevotella* and decreased of *Caulobacter* in BPD group at both times. *Prevotella* and *Caulobacter* were correlated with the severity of BPD (Spearman r = 0.551, r = − 0.545; *P* = 0.00005, 0.00006; respectively). Receiver operating characteristic analysis showed that the area under characteristic curve of *Caulobacter* model at first week reached 0.821 and *Prevotella* model at third week was 0.796. Moreover, microbial functional prediction analysis revealed that ABC-type transports were distinctively changed in BPD group. In summary, the use of non-invasive nasal swabs of microbiome to explore the pathophysiology in BPD is a compelling method worthy continuing to expand and research.

## Introduction

Bronchopulmonary dysplasia (BPD) is a chronic lung disease caused by mechanical ventilation and oxygen therapy and is the most common complication that affects premature infants^1^. The disease leads to longstanding consequences involving adverse effects on pulmonary function and neurodevelopmental outcome^2^. BPD rates are reported 40–55% in surviving extremely low gestational age neonates over the last few decades^3^. Despite the new advances in the field of neonatology, the incidence of BPD has largely been unchanged due to increased survival of extremely premature infants. There is still no ideal management for BPD disease and the only method to alleviate severity and improve the prognosis is early prevention. Therefore, early recognition of susceptible BPD is absolutely essential for timely intervention. Recent studies have demonstrated early airway microbiome may serve a role in modulating the infant's future susceptibility to severe BPD development^4^, suggesting another underlying pathway related to abnormal lung development^5–8^.

The airway microbiome can be identified early after birth and evolves over time with increasing bacterial loads and diversity^9^. Lal et al.^10^ were surprised to find that the airway microbiome of the neonates delivered by vaginal or cesarean section were similar, which indicated that the microbial DNA in the airway may be obtained through the placenta. They described the composition of the airway microbiota by analyzing the airway secretions on the first day of birth: *Firmicutes* and *Proteobacteria* were dominant and *Actinobacteria, Bacteroidetes, Tenericutes, Fusobacterium, Cyanobacteria*, and *Verrucomicrobia* were observed^10^. In BPD patients, as the disease progresses, the microbial community turnover increases, the relative abundance of *Proteobacteria* and *Firmicutes* changes, and *Lactobacillus* decreases^7^. Some important factors affect the composition and colonization of the pulmonary microbiota including prenatal and postnatal exposure to antibiotics, sepsis, environmental microbiome, mode of delivery, feeding and nutrition^11,12^. Study has also been reported the crosstalk between the lung and the intestine, which proposes a concept of the gut–lung axis, and the concomitant intestinal microbiota development also affects lung microbiota, resulting in pulmonary diseases^13^.

Pathogen detection requires sampling of lower airway secretions, which remains a challenge in non-expectorating patients. Currently, bronchoalveolar lavage (BAL) is considered as the gold standard; however, it cannot be performed and not suitable for every premature infant, so we explore a non-invasive nasal swab. The nasal cavities represent a highly accessible airway microbial community that recently was confirmed to have a pivotal role in human health and, to date, few studies focused on the microbiome of the nostrils of neonates^14,15^. Nasal cavity communicates with the outside world and is exposed to a variety of exogenous and endogenous microbes. It may play important roles in protecting against nasal colonization as well as invasive disease^16^. Previous studies have shown that there is a large amount of overlap between the nasal microbiota and the respiratory microbiota^16,17^, so the nasal microbiota could, to some extent, reflect the characteristics of the respiratory microbiota. Therefore, studying the composition and characteristic of nasal microbiota may open a window for exploring respiratory microbiota in preterm infants. The aim of this study is to: (1) assess the taxonomic composition of the early neonatal nasal swabs microbiota in BPD and control infants (2) probe potential underlying mechanisms and metabolic dysfunction in BPD disease.

## Results

### Clinical and sampling information for all infants

After applying inclusion and exclusion criteria, a total of 49 nasal swab samples were collected from 28 preterm infants, including 13 infants developed BPD and 15 controls were finally involved in this study in the NICU at Children’s Hospital, Zhejiang University School of Medicine, from 2019 to 2020 (Table 1). Three premature infants were transferred to the other units before collecting the second time specimens, so we didn’t get second nasal swabs. One baby died at 17th after birth due to respiratory failure. Gestational age ranged from 25^2/7^ to 30. Birth weights ranged from 700 to 1550 g. There are no significant differences between two groups in terms of gestational age, birth weight, gender, delivery mode, feeding, PDA, NEC, sepsis and antibiotics exposure (Table1, *P* > 0.05).

**Table 1:** Demographics of infants enrolled in the study.

	BPD (n = 13)	Control (n = 15)	*P*
Birth weight in g, median (range)	1125 (700–1480)	1120 (840–1550)	0.890
Gestational age in weeks, median (range)	28^4/7^ (25^2/7^–30)	29^4/7^ (25^4/7^–30)	0.267
Male gender, *n* (%)	7 (53.8)	8 (53.3)	0.978
Cesarean section, n (%)	8 (61.5)	8 (53.3)	0.662
Breast milk, n (%)	10 (76.9)	14 (93.3)	0.103
Rupture of membranes > 18 h, n (%)	1 (7.7)	3 (20.0)	0.353
NEC	0 (0.0)	1 (6.7)	0.343
PDA	10 (76.9)	8 (53.3)	0.194
Sepsis	1 (7.7)	3 (20.0)	0.353
Postnatal antibiotic exposure, -1w, median (range)	5 (0–8)	5 (0–7)	0.309
Postnatal antibiotic exposure, -3w, median (range)	5 (0–13)	7 (0–16)	0.068
**Severity of BPD**			
Grade 1	5	0	–
Grade 2	7	0	–
Grade 3	1	0	–
Grade 3A	1	0	
Invasive mechanical ventilation in days, median (range)	16 (0–73)	1 (0–18)	0.130
**Discharge outcomes**			
Death after 36 weeks PMA, n (%)	1 (7.7)	0 (0.0)	0.274
Tracheostomy placement, n (%)	0	0	–
Discharge on supplemental oxygen, n (%)	5 (5/11, 45.5)	0 (0.0)	0.004
Length of stay in days (mean ± SE)	82.0 ± 9.8	60.7 ± 4.4	0.065

*BPD* Bronchopulmonary dysplasia, *NEC* Necrotizing enterocolitis, *PDA* Patent ductus arteriosus.

### Microbial community characterization.

**Figure 1:**
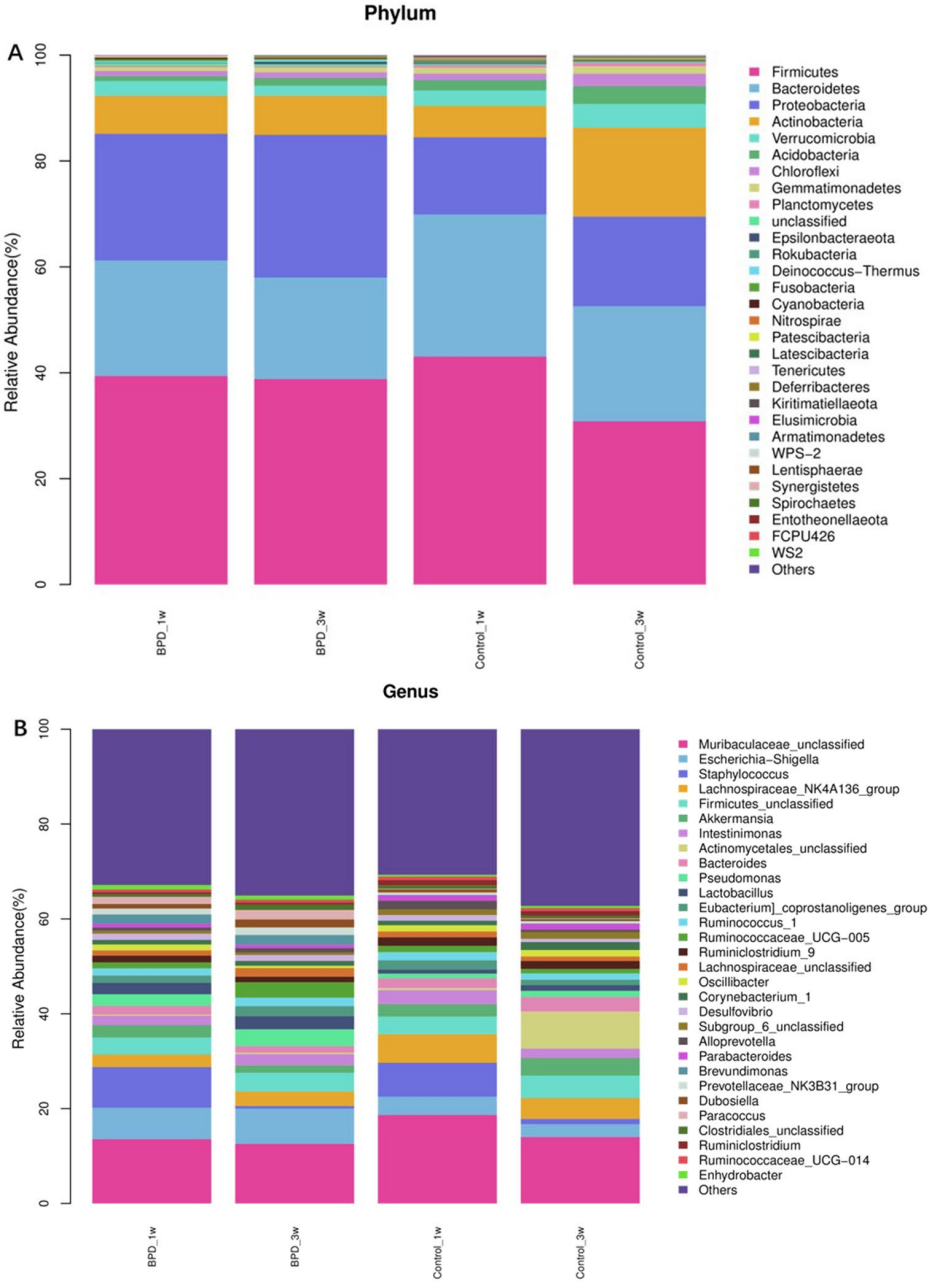
Gut microbiota composition and abundance in nasal swabs of preterm infants analyzed by 16S rDNA sequencing. (**A**) Composition of microbiota at the phylum level; (**B**) Composition of microbiota at the genus level. This figure was made using R version 3.5.2 (https://www.r-project.org/).

**Figure 2:**
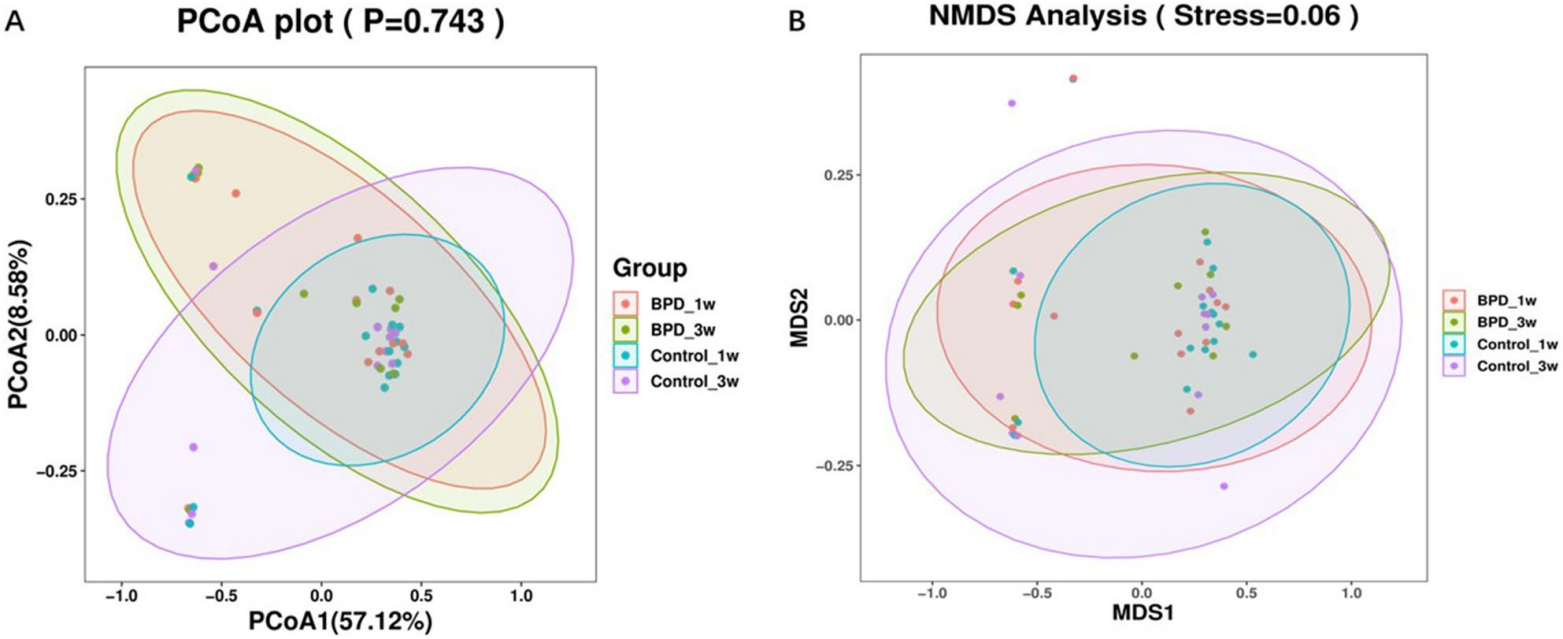
Beta diversity, each point in the figure represents a sample, and the points of the same color come from the same group. The closer the distance between the two points, the smaller the difference in community composition between the two points. The figure (**A**) shows the PCoA analysis results based on Weighted UniFrac, *P* values on the graph are derived from ANOSIM's calculations, *P* > 0.05. (**B**) Stress is an indicator reflecting the advantages and disadvantages of NMDS analysis results, Stress > 0.05. This figure was made using R version 3.5.2 (https://www.r-project.org/).

**Figure 3:**
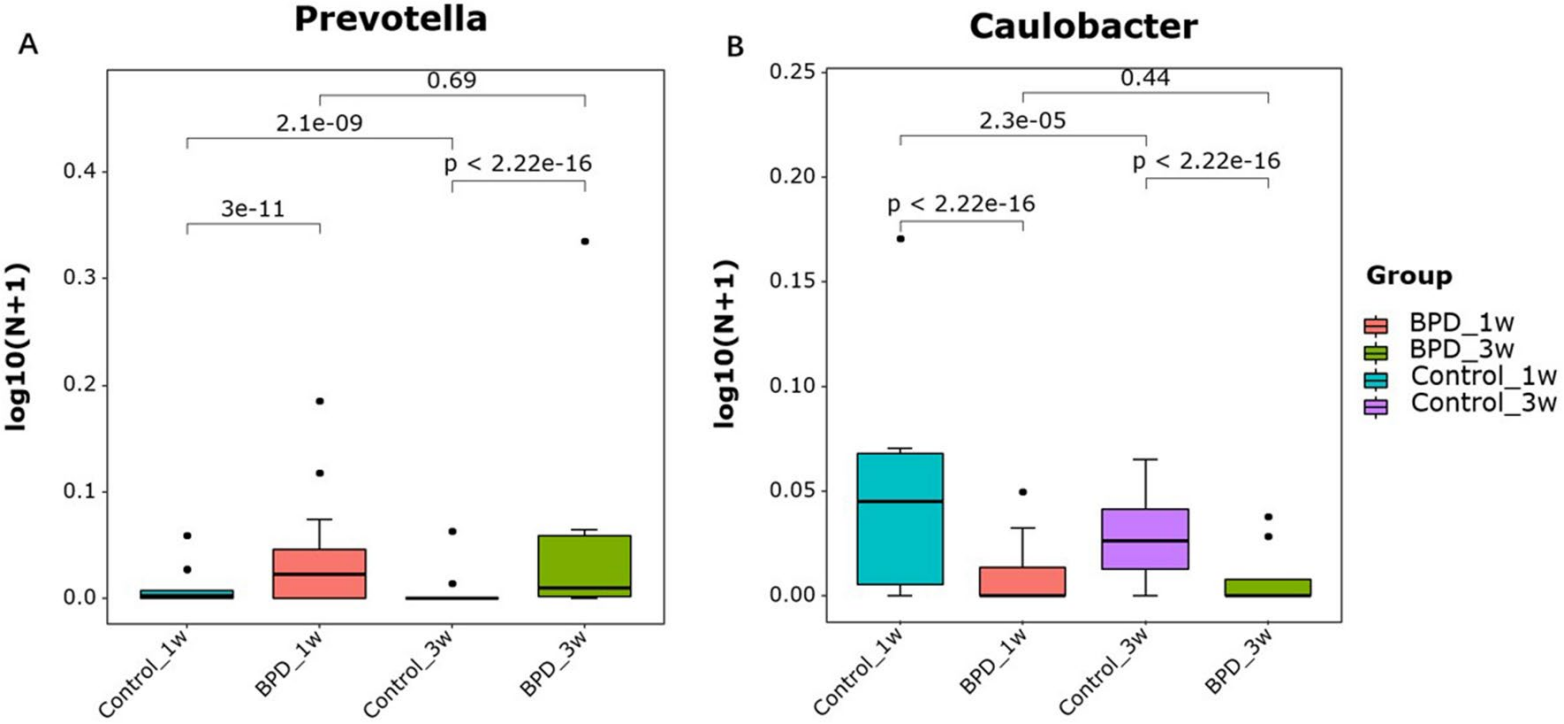
(**A**) Increased in the expression of *Prevotella* in BPD group at both time points (*P* < 0.05). (**B**) Decreased of *Caulobacter* in BPD group at both time points (*P* < 0.05). This figure was made using R version 3.5.2 (https://www.r-project.org/).

**Figure 4:**
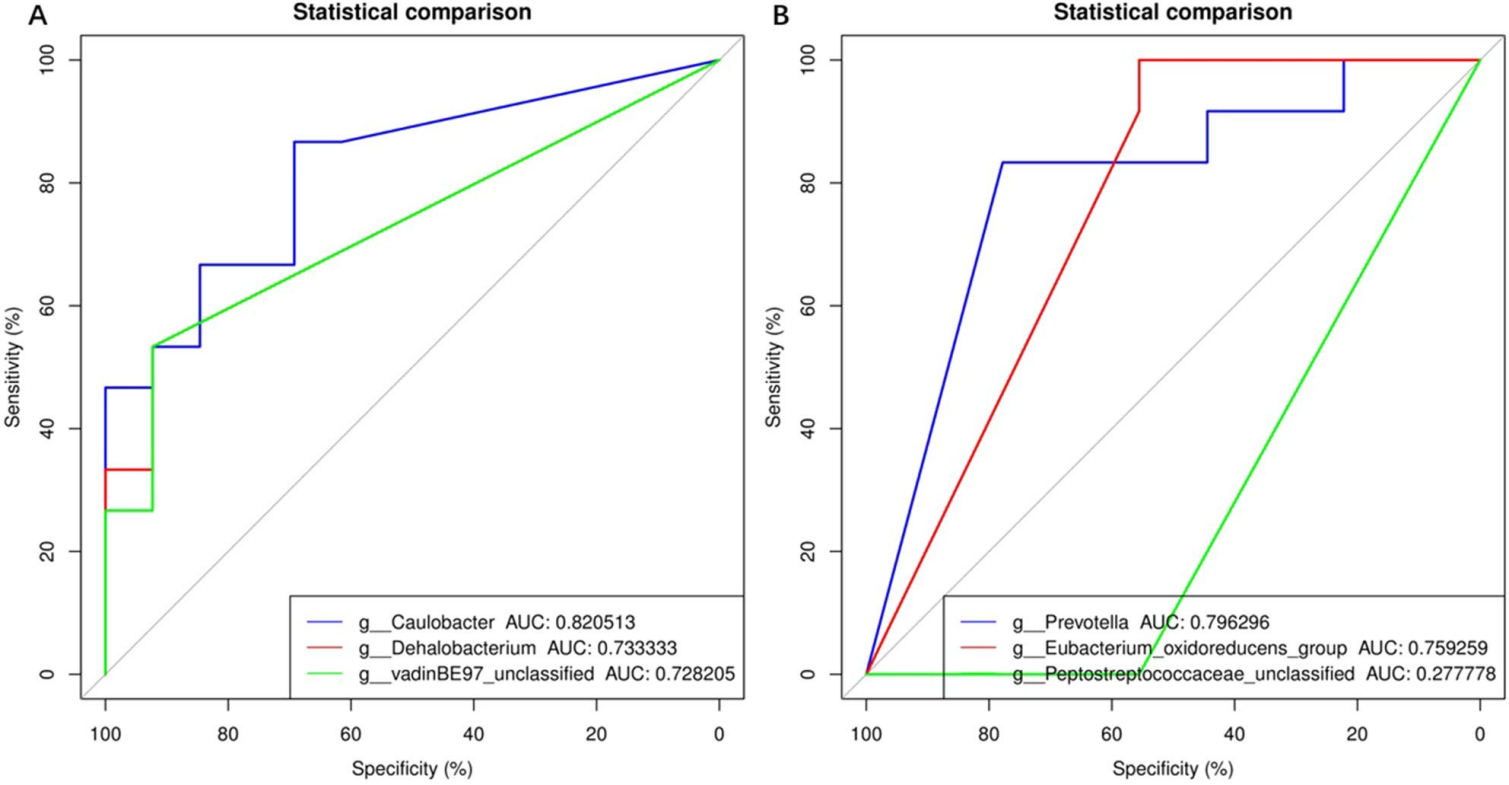
Receiver operating characteristic curves (ROC) for predicting BPD and top 3 AUC. (**A**) ROC at first week. curve blue *Caulobacter* AUC:0.821; curve red *Dehalobacterium* AUC:0.733; curve green *vadin BE97* AUC 0.728. (**B**) ROC at third week. curve blue Prevotella AUC:0.796; curve red Eubacterium AUC:0.759; curve green Peptostreptococcaceae AUC 0.278. This figure was made using R version 3.5.2 (https://www.r-project.org/).

Three samples had inadequate biomass for DNA sequencing and was excluded. A total of 2,951,645 high quality reads were obtained from the 49 samples, with a mean read count per sample of 60,238 (range 14,746–73,496). As shown in Fig. 1A, *Firmicutes, Bacteroidetes, Proteobacteria* and *Actinobacteria* were dominant phylums and shown in Fig. 1B, *Muribaculaceae, Escherichina, Staphylococcus* and *Lachnospiraceae* were dominant genus in all group. PCoA was performed to study the similarities or differences in sample community composition. NMDS analysis was performed using the weighted UniFrac distance algorithm, and two coordinate axes that could reflect the differences between samples to the greatest extent were selected for graphical display by dimension reduction of the multidimensional data. Based on the beta diversity, including PCoA and NMDS, no significant differences were detected in comparisons (*P* > 0.05, Fig. 2A,B). To evaluate which bacterial genera and species were involved in these observed temporal differences, we examined the relative abundances of the prevalent taxa. Some of the abundant genera and species changed significantly in relative abundance at first and third week (Kruskal–Wallis test, all *P*-values < 0.05) (Supplemental Figs. 1and 2). In summary, the species level differences between groups are often large and difficult to portray in graphic form. The genus level comparison is more amenable to presentation, which is one of the main values of 16S rDNA sequencing in the detection of organisms at the genus level. We present organism abundance data at the genus level to reduce complexity, given the larger number of species differentially abundant between Week 1 and Week 3 (species-level analysis revealed 11 and 17 species that were significantly different between Week 1 and Week 3 respectively with FDR < 0.05; Supplemental Tables  1and 2). We found increased in the expression of *Prevotella* and decreased of *Caulobacter* in BPD group at both time points (Fig. 3). We also found that *Prevotella* and *Caulobacter* were correlated with the severity of BPD (Spearman r = 0.551, r = − 0.545; *P* = 0.00005, 0.00006; respectively). The receiver operating characteristic (ROC) was drawn with the candidate genera, and the largest area under curve (AUC) was *Caulobacter* 0.821 at first week and *Prevotella* 0.796 at the third week respectively (Fig. 4). We also examined the contribution of invasive mechanical ventilation to microbiome composition at Weeks 1 and Weeks 3 in preterm infants and found diversity of the nasal microbiome were not significantly different between infant exposure to invasive mechanical ventilation compared with controls whereas some genera and species had a significant different expression. *Prevotella* was increased in invasive mechanical ventilation infants while *Caulobacter* was decreased both at week 1 and week 3 (Supplemental Fig. 3).

### Microbiota function prediction in BPD

**Figure 5:**
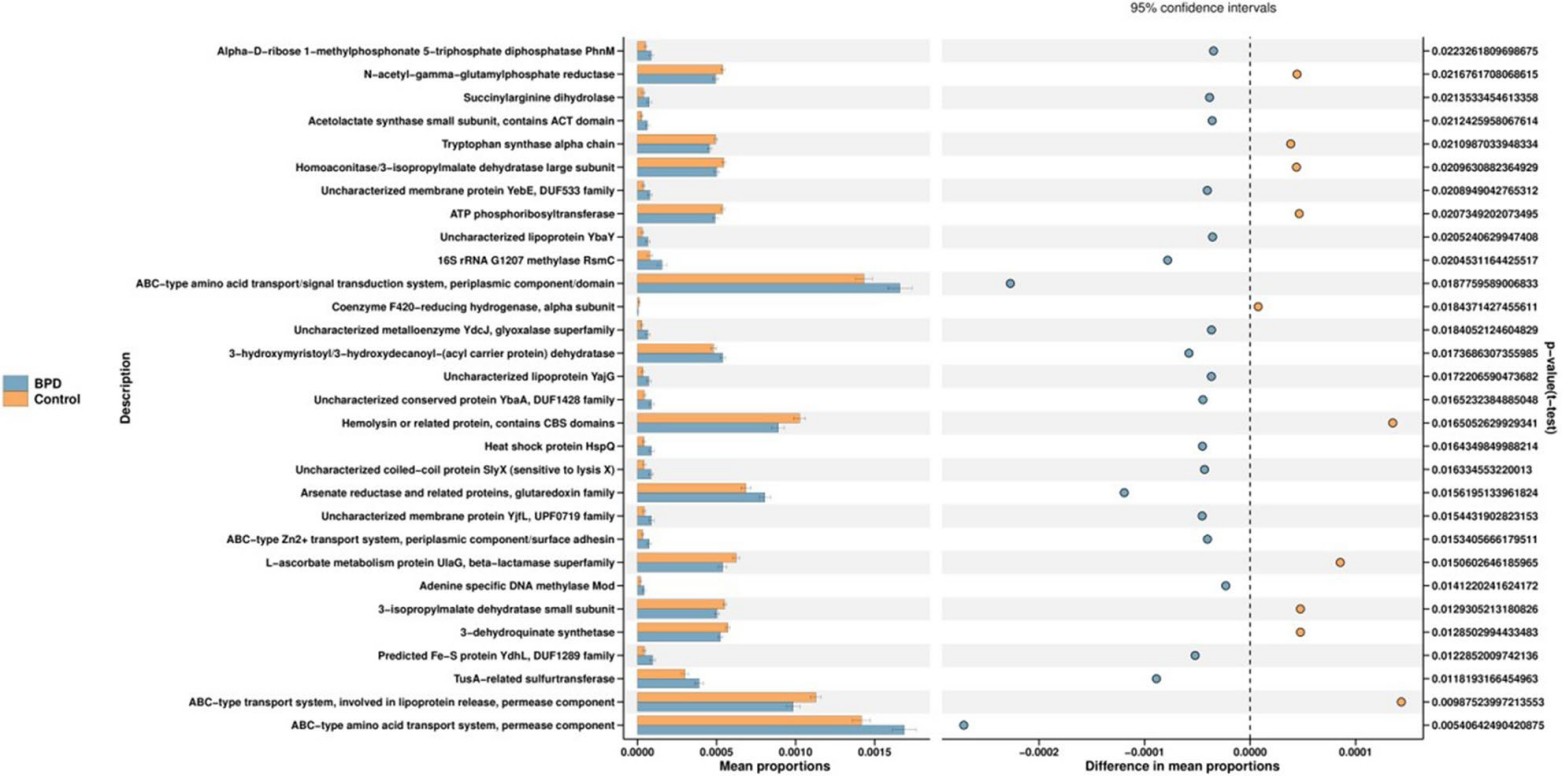
Microbiota function prediction in BPD. In the figure above, the COG database function annotation results of different grouping samples are compared, and the functions with significant differences between groups are screened out, where blue represents BPD group and orange represents control group. The horizontal bar chart on the left represents the percentages of all metabolic pathways enriched in the abundance of this metabolic pathway in the two groups of samples, and corrected p values are on the right. This figure was made using R version 3.5.2 (https://www.r-project.org/).

To infer metabolic pathways associated with the nasal taxa identified as differentially abundant based on BPD, we used PICRUSt (Phylogenetic Investigation of Communities by Reconstruction of Unobserved States) and STAMP (STatistical Analysis of Metagenomic Profiles)^18^ to map microbial genes to metabolic databases, in order to infer microbial functions differentially expressed by BPD. In order to discover the metabolic pathways and key proteins, we used the Clusters of Orthologous Groups of proteins function classification that have changed in BPD. The microbial functional prediction analysis revealed that ABC-type transport systems, transcription factors, amino acide, arginine/histidine, oligopeptide, spermidine/putrescine, taurine, thiamine and Zn2 + transport transport were distinctively increased in BPD group, while the ABC-type transport lipoprotein release was decreased in BPD group (Kruskal–Wallis, all *P* < 0.05) (Fig. 5).

## Discussion

Few studies have analyzed changes in the nasal microbiome in premature infants with BPD, providing a mechanistic explanation for changes in the microbiome during BPD and its impact on host-microbiome interactions. The present study of the nasal microbiome in BPD, we found no difference in nasal microbial diversity between preterm infants with BPD and controls. Our study identified *Firmicutes, Bacteroidetes, Proteobacteria* and *Actinobacteria* were dominant phylums and *Muribaculaceae, Escherichina, Staphylococcus* and *Lachnospiraceae* were dominant genus. We found increased in the expression of *Prevotella* and deceased of *Caulobacter* in BPD group at both time points. *Prevotella* and *Caulobacter* were correlated with the severity of BPD. Metagenomic prediction identified ABC-type transport systems metabolic pathways differentially associated with BPD.

In comparison to the gut microbiome, nasal microbiome has remained under studied. The nasal cavity serves as a physical transition from a space that is in constant contact with the external environment to an internal space. Within the nasal cavity, intra-generic interactions may lead to a different community structure at different microhabitats. In turn, these interactions may play important roles in protecting against nasal colonization as well as invasive disease^16,19^. Several studies revealed the microbiota developed in regions of the respiratory tract in newborns and during early life^15,20^. The nasal microbiota is of particular concern as the nostrils may harbor pathogens that can cause severe respiratory diseases^21^. Nasal microbiota compositions characterized by *Moraxella*, *Streptococcus*, or *Haemophilus* have been reported to be associated with upper respiratory infections^22^. Our study found that the expression of *Prevotella* was higher and *Caulobacter* was lower in the BPD group and it was correlated with the severity of BPD disease*.* Interestingly, bacterial *Prevotella* have been found to be prevalent commensal colonizers at mucosal sites; being the predominant genus in the respiratory system^23,24^. In light of the abundant *Prevotella* colonization and low pathogenicity, it is likely that humans have co‐evolved with *Prevotella.* However, emerging studies have linked increased *Prevotella* abundance and specific strains to inflammatory disorders, suggesting that at least some strains exhibit pathobiontic properties. Increased *Prevotella* abundance is associated with augmented Th17 mediated mucosal inflammation^25^. High *Prevotella* profile were associated with enhanced “subclinical” lung inflammation, with enhanced expression of inflammatory cytokines and elevated Th-17 lymphocytes^26^. In the study, we also found that *Prevotella* increased and *Caulobacter* decreased in infants with invasive mechanical ventilation, supporting the hypothesis of persistent lung inflammation after mechanical ventilation, especially *Prevotella* as a potential pathogen of early respiratory disease. Contrast to our study from tracheal aspirate samples were collected from 10 preterm infants with gestational age ≤ 34 weeks at birth who required mechanical ventilation, *Staphylococcus*, *Ureaplasma parvum*, and *Ureaplasma urealyticum* were the most frequently identified dominant organism^27^. Twenty-five infants, born at ≤ 32 week of gestation and intubated in the first 24 h, were enrolled and tracheal aspirates were obtained. They found that *Acinetobacter* was the predominant genus in the airways of all infants at birth^28^. Another study showed that genus *Lactobacillus* was decreased at birth in infants with chorioamnionitis and in preterm infants who subsequently went on to develop lung disease^10^. However, to clearly examine this, future in-depth studies of the preterm infant’s pulmonary microbiome are warranted and are ongoing. In addition, further research on the gut–lung axis and the potential role of nasal microbes in the development of respiratory disease needs to be explored.

The complementary functions of microbiome harbored inside and on the near mucosal surfaces of the host body are vital to maintain the host physiological homeostasis^29^. Recently, many studies report that in similar environment, the function of microbiome is also similar but the composition of microbiome might have great difference^30^. We found that ABC transporters which were responsible for the transportation and absorption of nutrients were significantly changed. ABC transporters are membrane proteins that are responsible for the uptake and secretion of a wide range of substrates, such as amino acids, sugars, xenobiotics, and vitamins up to polymers such as peptides, proteins, and polysaccharides^31^. ABC transporters have been implicated in a range of cellular processes, such as nutrition uptake, xenobiotic protection, extrusion of cellular waste products, bacterial immunity and virulence, osmotic stress, lipid transport, and differentiation^32^. We speculated that BPD changed the pH, oxygen tension, temperature of mucosal surfaces and this environment was not suitable for the living of predominant microbiome. Moreover, in our study, the amino acid transport regulated which was similar from previous studies. Ionescu et al.^33^ reports that protein catabolism increased in lung cystic fibrosis patients, probably due to the destruction of cellular and connective tissue proteins, which is related to the degree of impaired lung function and the systemic inflammatory response.

On the one hand, a limitation of this study is the small number of infants. A larger study population is needed to detect additional differences between subjects. On the other hand, although nasal swab to replace tracheal aspiration is a clinically relevant approach. Unfortunately, we did not compare the two kinds of samples. However, lower airway sampling is both ethically and technically challenging in prematurity. Importantly, analysis of microbiota during BPD in a larger number of infants is needed to understand the role of *Prevotella* and *Caulobacter*. Also, the causal and mechanistic pathways between *Prevotella*, *Caulobacter* and the microbiota metabolic pathways remain unclear. It needs to be assessed in translational approaches or using animal models. Given possible roles for noninvasive upper airway microbiota in BPD pathobiology, monitoring and investigation of BPD infants, the nasal microbiome in BPD is a compelling area of research to continue to expand.

## Materials and methods

### Recruited infants and sample collection

The study was performed as a prospective observational cohort design, and was approved by the ethics committee of the Children’s Hospital, Zhejiang University School of Medicine (2018-IRB-090-A2). Informed consent was obtained from at least one guardian of each patient and all procedures were conducted according to the guidelines. Preterm neonates less than or equal to 30 weeks of gestation were recruited from neonatal intensive care unit (NICU), Children's Hospital, Zhejiang University School of Medicine from 2019 to 2020. Exclusion criteria were major congenital anomalies of the lung or airway, known infection, or pneumonia. Sterile foam swabs were collected from anterior nares. Swabs were collected at 1 and 3 weeks of postnatal age. The first swab was collected by 5–7 days after birth following written informed consent from parents. The second swab was collected by 15–21 days of age. Swab tips were snapped off into sterile 1.5-ml polyethylene tubes, transferred immediately to − 80 °C freezer for storage. All infants were followed up until 36-week postmenstrual age, when the physiological definition of BPD. We used National Institute of Child Health and Human Development (NICHD) 2019 revision to define severity of BPD: no BPD, no support; grade 1 BPD, nasal cannula ≤ 2 L/min; grade 2 BPD, nasal cannula > 2 L/min or noninvasive positive airway pressure; and grade 3 BPD, invasive mechanical ventilation^1^. Grade 3A: early death (between 14 days of postnatal age and 36 weeks) owing to persistent parenchymal lung disease and respiratory failure that cannot be attributable to other neonatal morbidities (eg, necrotizing enterocolitis, intraventricular hemorrhage, redirection of care, episodes of sepsis, etc.)^34^. All infants were stratified into the following two groups: developed BPD (BPD group) or did not develop BPD (control group). Neonatal demographic data including gestational age, birth weight, gender, delivery mode, medication administration history, infants’ diet type (human milk vs. formula), significant events during NICU course and discharge outcomes were extracted from the electronic medical records.

### DNA extractions

DNA was extracted from swabs using the E.Z.N.A. ®Stool DNA Kit (D4015, Omega, Inc., USA) according to manufacturer’s instructions. The total DNA was eluted in 50 μL of Elution buffer and stored at − 80 °C until measurement.

### PCR amplification and 16S rDNA sequencing

The V3–V4 region of the bacterial small-subunit (16S) rRNA gene was amplified with primers 341F (5′-CCTACGGGNGGCWGCAG-3′) and 805R (5′-GACTACHVGGGTATCTAATCC-3′)^35^. PCR amplification was performed in a total volume of 25 μL reaction mixture containing 25 ng of template DNA, 12.5 μL PCR Premix, 2.5 μL of each primer. The PCR conditions is initial denaturation at 98 °C for 30 s; 32cycles of denaturation at 98 °C for 10 s, annealing at 54 °C for 30 s, and extension at 72 °C for 45 s; and then final extension at 72 °C for 10 min. The PCR products were purified by AMPure XT beads (Beckman Coulter Genomics, Danvers, MA, USA) and quantified by Qubit (Invitrogen, USA). The amplicon pools were prepared for sequencing and the size and quantity of the amplicon library were assessed on Agilent 2100 Bioanalyzer (Agilent, USA) and with the Library Quantification Kit for Illumina (Kapa Biosciences, Woburn, MA, USA), respectively. Samples were sequenced on an Illumina NovaSeq platform according to the manufacturer's recommendations (LC-Bio Technology Co., Ltd, Hang Zhou, China).

### Data analysis

Paired-end reads was assigned to samples based on their unique barcode and truncated by cutting off the barcode and primer sequence. Paired-end reads were merged using FLASH Quality filtering on the raw reads were performed under specific filtering conditions to obtain the high-quality clean tags according to the fqtrim (v0.94). Chimeric sequences were filtered using Vsearch software (v2.3.4). After dereplication using DADA2, we obtained feature table and feature sequence. Principal coordinate analysis (PCoA) analysis was displayed by QIIME2 and ggplot2 package. Nonmetric multidimensional scaling (NMDS) analysis was performed with the vegan package and displayed with the ggplot2 package in R software. The predictive power was evaluated by the ROC curve and the AUC. Cut-off values of the results were chosen on the basis of the ROC curve of each variable, which located in the maximum value of Youden Index according to the sensitivity and specificity. AUC was used to analyze the disease diagnosis ability of the candidate biomarkers selected based on the results. The figures were drawn by R (v3.5.2).

## Supplementary Information

Supplementary Information 1.

Supplementary Information 2.

Supplementary Information 3.

Supplementary Information 4.

Supplementary Information 5.

Supplementary Information 6.

## Data Availability

Data are available in a public, open access repository. Sequence data have been deposited to the NCBI Sequence Read Archive and are available under accession number PRJNA782204.
