# Supplementary figures and images for: The nasal microbiome of predicting bronchopulmonary dysplasia in preterm infants
Source: Sci Rep. 2022 May 11;12:7727. doi: 10.1038/s41598-022-10770-3 (PMC9095869; doi:10.1038/s41598-022-10770-3)

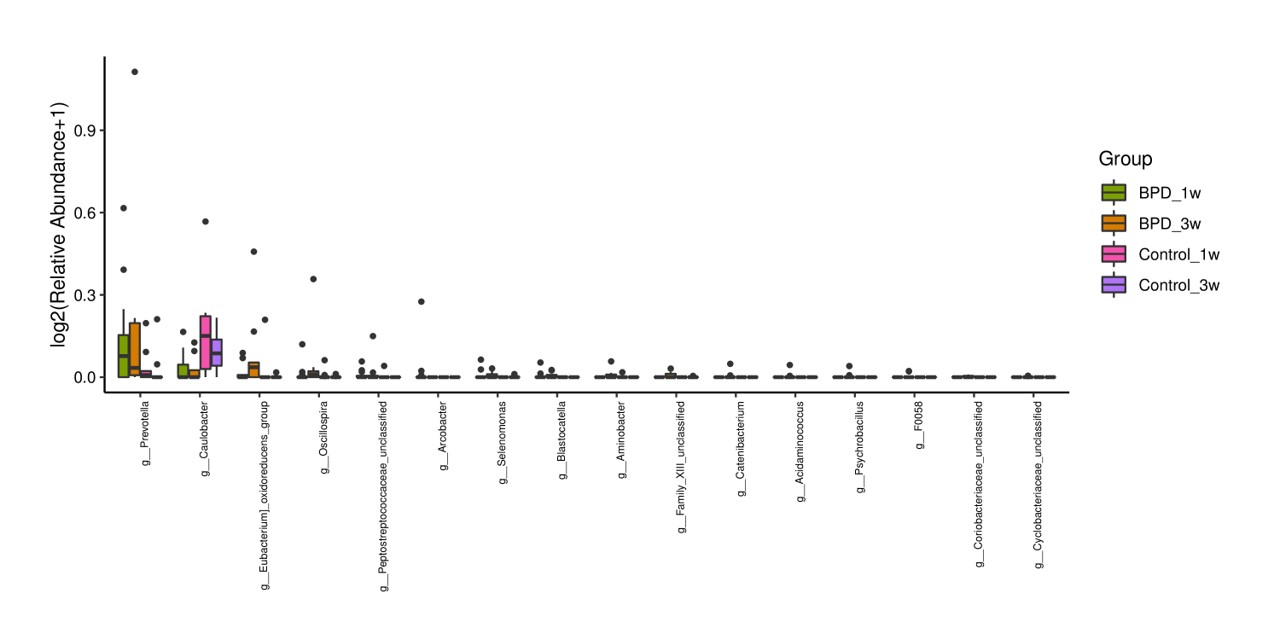

Supplement: Supplementary file 1 — Supplementary Information 1. [file 41598_2022_10770_MOESM1_ESM.jpg]

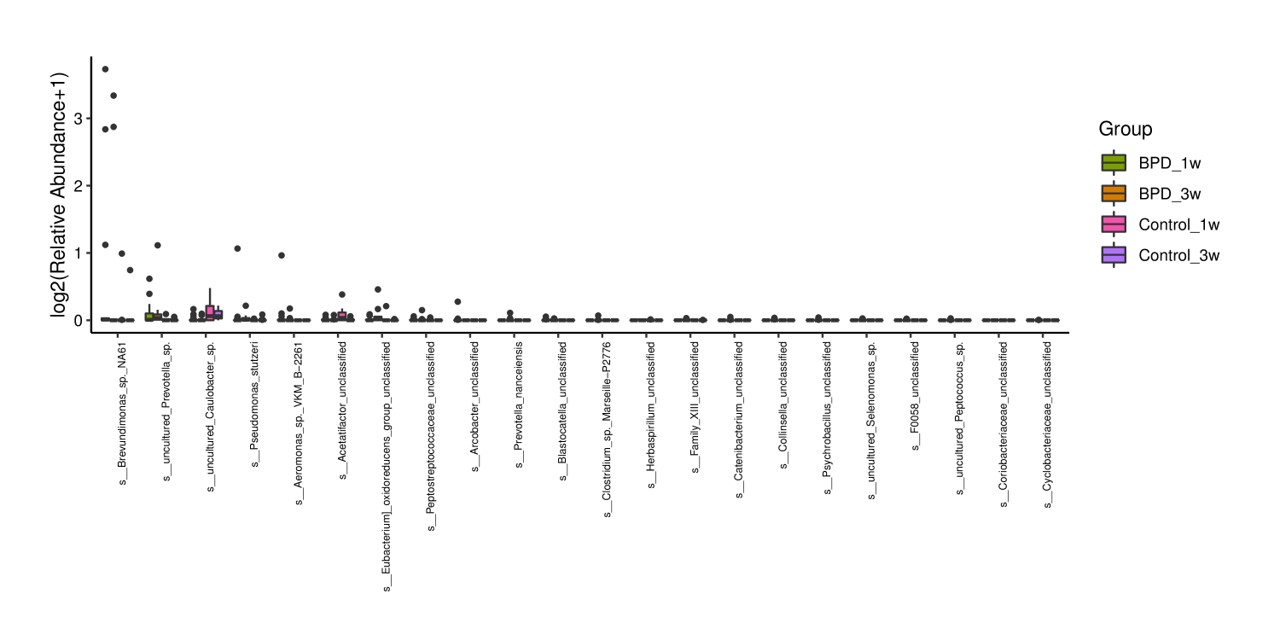

Supplement: Supplementary file 2 — Supplementary Information 2. [file 41598_2022_10770_MOESM2_ESM.jpg]

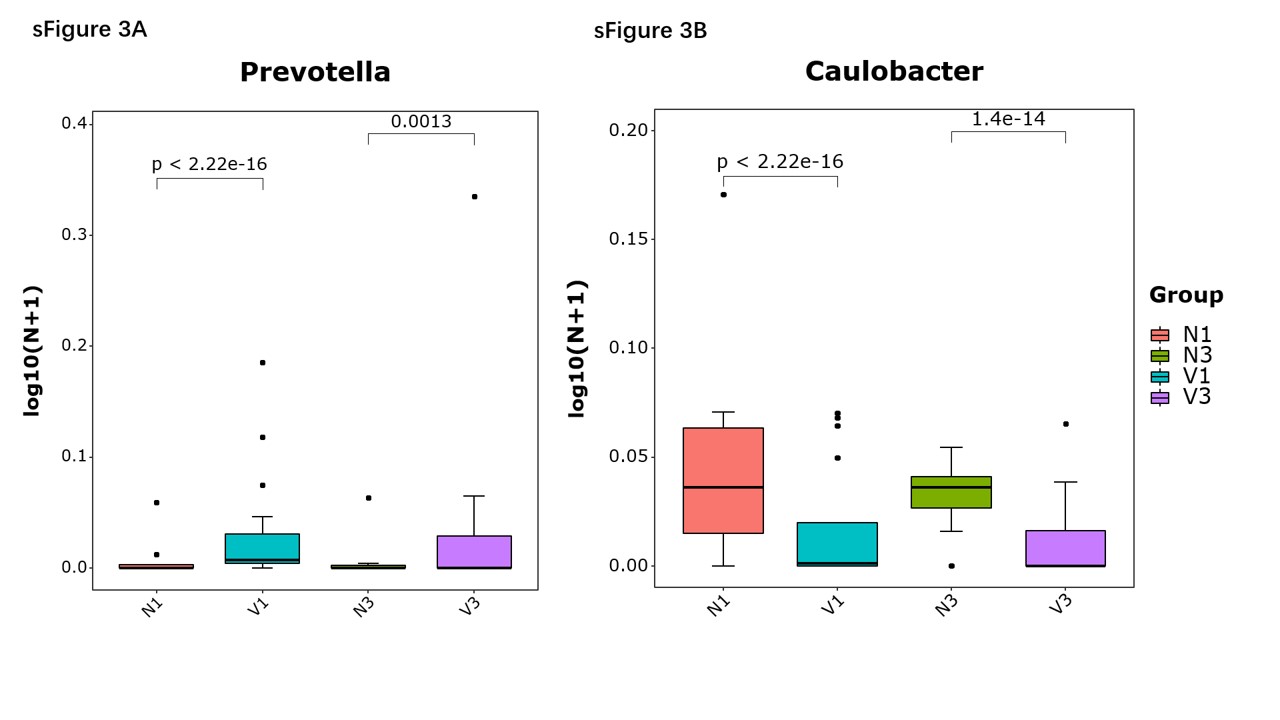

Supplement: Supplementary file 3 — Supplementary Information 3. [file 41598_2022_10770_MOESM3_ESM.jpg]
