# Supplementary material for: The nasal microbiome of predicting bronchopulmonary dysplasia in preterm infants
Source: Sci Rep. 2022 May 11;12:7727. doi: 10.1038/s41598-022-10770-3 (PMC9095869; doi:10.1038/s41598-022-10770-3)
Supplement: Supplementary file 4 — Supplementary Information 4. [file 41598_2022_10770_MOESM4_ESM.docx]

**Supplemental Figure legends**

**Supplemental Figure 1** The results included significant differences in microbiota at the genera level and in different comparison groups. P<0.05 defined as significantly different. This figure was made using R version 3.5.2 (https://www.r-project.org/).

**Supplemental Figure 2** The results included significant differences in microbiota at the species level and in different comparison groups. P<0.05 defined as significantly different. This figure was made using R version 3.5.2 (https://www.r-project.org/).

**Supplemental Figure 3** (A) Increased in the expression of *Prevotella* in invasive mechanical ventilation group at both time points (p<0.05). (B) Decreased of *Caulobacter* in invasive mechanical ventilation group at both time points (p<0.05). V1= invasive mechanical ventilation group at first week, N1=control group at first week, V3= invasive mechanical ventilation group at third week, N3=control group at third week. This figure was made using R version 3.5.2 (https://www.r-project.org/).

**Supplemental Table legends**

**Supplemental Table 1** The relative abundance of the microbiome at the species level was in week 1.

**Supplemental Table 2** The relative abundance of the microbiome at the species level was in week 3.
